# Mental health, smoking, harm reduction and quit attempts – a population survey in England

**DOI:** 10.1186/s12889-020-09308-x

**Published:** 2020-08-14

**Authors:** Leonie S. Brose, Jamie Brown, Debbie Robson, Ann McNeill

**Affiliations:** 1 Addictions, Institute of Psychiatry, Psychology and Neuroscience, King’s College London, 4 Windsor Walk, London, SE5 8BB UK; 2 SPECTRUM Consortium, UK; 3 Research Department of Clinical, Educational and Health Psychology, University College London, 1-19 Torrington Place, London, WC1E 7HB UK; 4 Department of Epidemiology and Public Health, University College London, 1-19 Torrington Place, London, WC1E 7HB UK

**Keywords:** Tobacco smoking, Smoking cessation, Mental health, Electronic cigarettes, Harm reduction

## Abstract

**Background:**

Tobacco control strategies have engendered overall declines in smoking; however, a large gap remains between people with and without mental health problems, causing substantial health inequalities. Population-level information on barriers and opportunities for improvements is scarce. We aimed to assess mental health status of cigarette smokers and recent ex-smokers (‘past-year smokers’) in England, and smoking and harm reduction behaviour and quit attempts by mental health status.

**Methods:**

Data were collected from 5637 current and 434 recent ex-smokers in 2016/17 in household surveys of representative samples of adults. We calculated weighted prevalence of different indicators of mental health problem: a) ever diagnosis, b) none, moderate, serious past-month distress, c) past-year treatment. We compared weighted smoking status, cigarette type, dependence, motivation to stop smoking, cutting down, use of nicotine replacement therapy or e-cigarettes, short-term abstinence, and quit attempts according to mental health status.

**Results:**

Among past-year smokers: 35.9% ever had a diagnosis; 24.3% had experienced moderate, an additional 9.7% serious, past-month distress; 21.9% had had past-year treatment. Those with an indication of a mental health problem were more highly dependent and more likely to smoke roll-your-own cigarettes but also more likely to be motivated to stop smoking, to cut down, use nicotine replacement therapy or e-cigarettes and to have attempted to quit in the past year.

**Conclusions:**

About a third of cigarette smokers in England have mental health problems. Interventions should address their increased dependence and leverage higher prevalence of harm reduction behaviours, motivation to stop and attempts to stop smoking.

## Background

There are substantial inequalities in morbidity and premature mortality between individuals with mental health problems and those without and much of this inequality is due to smoking [1–3]. Tobacco control strategies have led to an overall decline in smoking prevalence in countries such as the UK and US, however, there remains a large gap in smoking prevalence between people with and without mental health problems. Among those with a common mental health disorder in England, smoking prevalence remains around 50% higher [4] and this increases further for more severe mental disorders [5]. Figures for the US similarly show an increased burden of smoking among those with mental health problems [6, 7]. The relationship is complex but appears to be bidirectional [8–10].

Previous research has consistently shown that smokers with mental health problems on average smoke more heavily [4, 5, 7] and extract more nicotine from each cigarette than those without mental health problems [5]. People with schizophrenia, bipolar disorder, depression and anxiety who smoke experience more severe symptoms and require higher doses of some psychotropic medicines than non-smokers [5]. Smoking is also associated with poorer treatment outcomes; compared with non-smokers with mental health problems, smokers with mental health problems spend longer time in hospital and less time out of hospital [11]. There is some evidence, mainly from the US, that smokers with mental health problems are as likely to make a quit attempt as other smokers [12] but cessation rates are lower. Successful quitting is associated with improved mental health, specifically reduced anxiety, depression and stress and increased psychological quality of life and positive affect [13]; additionally, for specific medications for severe mental illness, quitting may reduce the required dosage [5] and thus associated side effects.

Preventing the uptake of smoking, prompting more quit attempts and improving access to smoking cessation or harm reduction interventions are needed to reduce the excess burden of disease and premature mortality associated with smoking among people with mental health problems. Research about the effectiveness of smoking cessation interventions among smokers with severe mental health problems is promising [14, 15]. However, advancement of smoking cessation for smokers with mental health problems is limited, particularly in England and the rest of the UK, by a lack of population level studies about smoking prevalence, motivation to quit, attempts to quit and harm reduction behaviours which are needed to quantify the need and identify barriers and opportunities for interventions.

This study aimed to answer the following questions:

1. What is the mental health status of current cigarette smokers and recent ex-smokers (‘past-year smokers’) in England?
2. What are the characteristics of smoking behaviour among past-year smokers with and without mental health problems?
3. What are the characteristics of harm reduction behaviour among current cigarette smokers with and without mental health problems?
4. What is the frequency of quit attempts in past-year smokers with and without mental health problems?

## Methods

### Procedure

The present study uses self-reported data from monthly cross-sectional household surveys of representative samples of the population of adults in England collected as part of the ongoing Smoking and Alcohol Toolkit Study. The methodology for the Toolkit Study has been described in detail by Fidler et al. [16] and to date, it has been used as the basis for approximately 85 peer-reviewed publications. Briefly, each month (wave), a new sample of approximately 1700 adults aged 16 years or older in England completes a face-to-face computer-assisted survey. After stratification by geo-demographic classification of population, small geographical areas containing approximately 300 households are allocated randomly to interviewers who visit households within the locality and conduct computer-assisted face-to-face interviews with one member of a household in those areas until a pre-specified quota tailored to the area is fulfilled. This form of sampling has benefits over conventional quota sampling, because the allocation of small areas to interviewers reduces the impact of selection bias resulting from the selection of properties [17]. Response rates cannot be calculated because of the lack of a definitive gross sample: all units fulfilling the criteria of a given quota are interchangeable within the areas. For the present study, measures of mental health were added to the survey for 24 monthly waves from January 2016 to December 2017.

### Measures

All measures were self-reported. Generally, questions were completed in an interview with the interviewer recording the responses; the mental health questions were self-completed by the interviewee on a laptop following familiarisation using similar example questions.

*Sociodemographic measures* included age (16–24; 25–34; 35–44; 45–54; 55–64; 65 and over), gender and occupational grade (coded AB for higher or intermediate managerial, administrative or professional occupation; C1 for Supervisory or clerical and junior managerial, administrative or professional; C2 for skilled manual workers; D for semi-skilled and unskilled manual workers; E for state pensioners, casual and lowest grade workers, unemployed with state benefits only [18]).

*Smoking-related measures* included smoking status, assessed using “Which of the following best applies to you? Please note we are referring to cigarettes and other kinds of tobacco that you set light to and NOT electronic or ‘heat-not-burn’ cigarettes. a) I smoke cigarettes (including hand-rolled) every day; b) I smoke cigarettes (including hand-rolled), but not every day; c) I do not smoke cigarettes at all, but I do smoke tobacco of some kind (e.g. pipe, cigar or shisha); d) I have stopped smoking completely in the last year; e) I stopped smoking completely more than a year ago; f) I have never been a smoker (i.e. smoked for a year or more).” Those who had stopped smoking more than a year ago (response e) or had never been smokers (f) were excluded as were the 186 smokers who reported currently smoking other tobacco products (c). Those selecting responses a or b were categorised as current smokers; current smokers and those who had stopped in the last year (d) were categorised as past-year smokers. Those who had stopped smoking may have included a small number of respondents who had smoked products other than cigarettes. Past-year cigarette smokers reported type of cigarette smoked (hand-rolled, manufactured or both), strengths of urges to smoke [19] and heaviness of smoking index (cigarettes per day and time to first cigarette [20]); current cigarette smokers also reported motivation to stop smoking [21] and average spend on tobacco per week.

*Harm reduction measures* for all past-year smokers included number of attempts to quit smoking in the past year and whether abstinence from smoking of one month or longer was achieved during the past year which was derived from questions about how long each of up to three quit attempts undertaken in the last year lasted before going back to smoking. Current smokers were additionally asked whether they were currently attempting to cut down on the number of cigarettes and whether they were currently using nicotine replacement therapy (NRT) or e-cigarettes/vaping products (EC) for cutting down, temporary abstinence or other reasons.

*Mental health measures* included three indicators of mental health problems:

1. Ever diagnosis: Participants were asked: “Since the age of 16, which of the following, if any, has a doctor or health professional ever told you that you had? Depression; Anxiety; Obsessive Compulsive Disorder; Panic Disorder or a phobia; Post-traumatic Stress Disorder; Psychosis; Personality Disorder; Attention Deficit Hyperactivity Disorder; An Eating Disorder; Alcohol Misuse or Dependence; Drug Use or Dependence; Problem Gambling; None of these; Don’t know; Prefer not to say.” Question wording was adapted from previous surveys [22, 23], the list of diagnoses based on a report on adult psychiatric morbidity in England [24]. Response options excluding the final three were presented in a randomised order. Individual diagnoses were dummy-coded. For analysis, a composite measure of ‘Any diagnosis’ was derived.
2. Past-month distress: Measured using the K6 screener for mental distress in the past 30 days [25, 26]: “During the past 30 days, about how often, if at all, did you feel … nervous; hopeless; restless or fidgety; so depressed that nothing could cheer you up; that everything was an effort; worthless?” The options were presented in a randomised order and for each the respondent indicated one of the following: “All of the time (scored 4); Most of the time [3]; Some of the time [2]; A little of the time [1]; None of the time (0)”; these options were presented in this or the reverse order for the entire K6. Additional response options “Don’t know; Prefer not to say” were provided. The K6 has been validated and used in a number of population surveys [25–35]. A sum score with a possible range from 0 to 24 was calculated and following common practice, scores of 13 and higher categorised as serious distress [25, 29]. Some publications have additionally used a category of moderate distress (scores 5–12) [31] which was also used.
3. Past-year treatment: Respondents who selected any of the responses for ever diagnosis were asked for those they selected: “In the last 12 months, which of the following conditions, if any, have you had any treatment or taken any prescribed medication for?”.

### Sample selection

In 2016 and 2017, 40,831 adults were surveyed of whom 7651 were past-year (current and recent-ex) smokers who were asked the mental health questions. Smokers who exclusively smoked tobacco products (pipes, cigars) other than cigarettes were excluded (*n* = 186). Those who did not complete the mental health questions or selected ‘don’t know’ or ‘prefer not to say’ in response to any of them were also excluded, leaving 6280 past-year smokers with information on mental health. Finally, those with missing data on any of the other variables included in the present analysis were excluded, leaving 6071 past-year smokers for the main analyses, of whom 5637 were current cigarette smokers and 434 recent ex-smokers. Type of cigarette smoked was missing for 164, reducing the sample to 5907 past-year smokers and 5506 current cigarette smokers if type of cigarette was included in analysis.

### Analysis

Past-year smokers who completed the mental health information were compared with those who did not complete the mental health information or responded don’t know or prefer not to say.

To address research question 1 (mental health), weighted proportions were used to describe the prevalence of ever having had any mental health diagnosis since the age of 16, the prevalence of moderate and serious distress in the past month and the prevalence of treatment for mental health problems in the past year overall and by diagnosis.

To address research question 2 (smoking behaviour by mental health), weighted proportions were calculated and chi-square statistics were used to compare smoking status, type of cigarette smoked, dependence (as measured using urges to smoke and heaviness of smoking) of past-year cigarette smokers with and without any diagnosis, with minimal, moderate and serious past-month distress and with and without past-year treatment. For chi-square tests, Cramer’s V was used as effect size. Cramer’s V can range from 0 (no association) to 1 (perfect association). Additionally, for current cigarette smokers, spend on tobacco was compared using analyses of variance (ANOVAs) and independent samples median tests.

To address research question 3 (harm reduction behaviour), weighted proportions for cutting down, using e-cigarettes, using NRT, using either e-cigarettes or NRT, motivation to stop smoking and having achieved at least one month of abstinence in the past year were calculated for current cigarette smokers and compared using chi-square statistics with Cramer’s V as effect size.

To address research question 4 (quit attempts), the mean number of quit attempts made in the past year was compared among past-year smokers with and without mental health problems. A dichotomised version of this measure (at least one quit attempt versus none) was used to calculate prevalence and 95% confidence intervals of having made at least one quit attempt and as outcome in logistic regressions assessing its association with ever diagnosis, past-month levels of distress and past-year treatment among past-year smokers. Unadjusted models were followed by models adjusting for socio-demographics. Additional models adjusted for motivation to stop smoking and type of cigarette; as this information can only be collected from current cigarette smokers, any quit attempt had been unsuccessful.

The analyses plan was pre-registered on the Open Science Framework (https://osf.io/9pbv8/). The following changes were made from the registration: Research question 1 was added to provide a fuller description of the population. The present research questions 2 and 3 were initially presented as a single research question. To increase consistency, smokers of other tobacco products such as pipes or cigars were excluded from all analysis.

## Results

Non-completion of mental health information became slightly more likely with increasing age and was more likely for non-daily cigarette smokers and current NRT users, but differences were small (Supplementary file 1, Table S1).

### Mental health

Overall, 35.9% of past-year smokers reported ever having been diagnosed with at least one mental health problem since the age of 16, 34.0% reported having experienced moderate (24.3%) or serious distress (9.7%) in the past month and 21.9% of the sample had received treatment for a mental health in the past year. Among those who had ever been diagnosed with a mental health problem, the most common diagnosis was depression, followed by anxiety (Supplementary file 2, Table S2). In the past year, 61.0% of those with a diagnosis had received treatment for this diagnosis, however, this ranged from 7.9% for problem gambling to 64.1% for psychosis (Supplementary file 2, Table S2). In general, indicators of mental health problems were more common among younger age groups, women and lower socio-economic groups (Table 1).

**Table 1:** Sample description (weighted data) by indicator of mental health problem in past-year smokers and the subset of current smokers

		All	Ever diagnosis			Past-month distress				Past-year treatment		
Past-year cigarette smokers			No	Yes	Test statistics	Minimal	Moderate	Serious	Test statistics	No	Yes	Test statistics
Unweighted n		6071	3904	2167		3981	1499	591		4742	1329	
**Age, %**	16–24	18.8	18.4	19.6	χ^2^ = 87.89, p < 0.001, V = 0.118	15.1	25.9	26.1	χ^2^ = 207.65, *p* < 0.001, V = 0.128	18.7	19.2	χ^2^ = 74.83, *p* < 0.001, V = 0.109
	25–34	22.5	21.7	24.1		21.8	23.7	24.6		22.2	23.7	
	35–44	18.2	17.1	20.0		17.6	19.2	19.2		17.3	21.3	
	45–54	18.3	17.7	19.5		19.3	15.7	18.1		17.6	20.9	
	55–64	11.9	12.4	11.2		13.4	9.0	9.6		12.4	10.2	
	≥65	10.2	12.8	5.7		12.8	6.5	2.3		11.8	4.7	
**Gender, %** ^**a**^	Men	52.4	57.6	43.0	χ^2^ = 124.73, p < 0.001, V = 0.141	55.5	48.2	41.2	χ^2^ = 58.32, p < 0.001, V = 0.096	56.2	38.7	χ^2^ = 132.23, p < 0.001, V = 0.145
	Women	47.6	42.4	57.0		44.5	51.8	58.8		43.8	61.3	
**Occupational grade, %**	AB	14.9	16.0	13.1	χ^2^ = 209.54, p < 0.001, V = 0.182	16.3	13.2	10.4	χ^2^ = 235.35, p < 0.001, V = 0.136	15.8	11.7	χ^2^ = 253.20, p < 0.001, V = 0.200
	C1	24.5	25.1	23.5		24.9	25.2	20.4		25.4	21.5	
	C2	25.7	27.8	21.9		27.7	23.0	18.6		27.3	20.0	
	D	20.2	21.2	18.3		20.7	18.7	19.9		20.5	18.8	
	E	14.7	9.9	23.2		10.4	19.9	30.7		11.0	27.9	
**Smoking, %**	Daily cigs	81.6	82.0	81.0	χ^2^ = 3.58, *p* = 0.167, V = 0.024	82.7	78.8	82.1	χ^2^ = 11.44, *p* = 0.022, V = 0.030	81.6	81.8	χ^2^ = 8.60, *p* = 0.014, V = 0.037
	Non-daily cigs	10.9	11.0	10.7		10.3	12.6	10.8		11.3	9.3	
	Stopped last year	7.5	7.0	8.3		7.0	8.7	7.2		7.1	8.8	
**Type of cigarette, %** ^**b**^	Manufactured	50.4	56.2	40.2	χ^2^ = 146.15, p < 0.001, V = 0.154	55.3	43.6	34.1	χ^2^ = 131.67, *p* < 0.001, V = 0.103	53.6	39.3	χ^2^ = 87.92, *p* < 0.001, V = 0.120
	Roll-your-own	44.1	38.8	53.7		40.0	50.1	57.8		41.5	53.6	
	Mix	5.4	5.0	6.1		4.7	6.3	8.1		4.9	7.2	
**Urges to smoke, %**	None	16.9	18.9	13.5	χ^2^ = 117.41, *p* < 0.001, V = 0.136	18.4	14.8	12.6	χ^2^ = 179.27, p < 0.001, V = 0.12	18.2	12.3	χ^2^ = 111.32, p < 0.001, V = 0.133
	1	16.3	17.4	14.4		17.7	14.4	11.7		17.0	13.8	
	2	43.3	43.6	42.6		44.1	43.6	36.7		43.7	41.9	
	3	17.3	15.8	20.0		15.6	19.2	24.0		16.2	21.1	
	4	4.5	3.4	6.4		3.2	5.6	10.4		3.6	7.5	
	5	1.7	0.9	3.0		1.0	2.3	4.6		1.2	3.4	
**HSI, %**	Low (< 4)	87.5	90.4	82.4	χ^2^ = 84.35, p < 0.001, V = 0.116	89.8	85.2	77.7	χ^2^ = 82.78, p < 0.001, V = 0.114	89.4	80.7	χ^2^ = 75.31, p < 0.001, V = 0.109
	High (4+)	12.5	9.6	17.6		10.2	14.8	22.3		10.6	19.3	
**≥1 quit attempt, %** ^**b**^	Yes	32.5	30.4	36.2	χ^2^ = 22.00, p < 0.001, V = 0.059	29.6	37.7	38.8	χ^2^ = 46.28, p < 0.001, V = 0.086	30.8	38.3	χ^2^ = 27.05, p < 0.001, V = 0.065
**Abstinence ≥ 1 month, %**	Yes	12.9	12.3	13.8	χ^2^ = 3.01, *p* = 0.083, V = 0.022	11.6	16.1	13.4	χ^2^ = 20.39, p < 0.001, V = 0.057	12.5	14.1	χ^2^ = 2.40, *p* = 0.122, V = 0.019
**Current cigarette smokers**												
**Unweighted n**		5637	3646	1991		3708	1381	548		4420	1217	
**MTSS %**	In ≤3 months	14.4	13.3	16.3	χ^2^ = 9.79, *p* = 0.002, V = 0.041	13.0	17.7	15.6	χ^2^ = 19.30, p < 0.001, V = 0.057	13.6	17.3	χ^2^ = 11.15, *p* = 0.001, V = 0.044
**Cutting down, %**	Yes	46.1	43.1	51.4	χ^2^ = 37.12, p < 0.001, V = 0.080	42.8	52.2	53.3	χ^2^ = 49.39, p < 0.001, V = 0.092	44.5	52.0	χ^2^ = 22.57, p < 0.001, V = 0.062
**Current NRT, %**	Yes	8.5	7.5	10.5	χ^2^ = 15.60, p < 0.001, V = 0.052	7.6	10.0	11.2	χ^2^ = 13.33, p = 0.001, V = 0.048	7.6	11.8	χ^2^ = 22.26, p < 0.001, V = 0.062
**Current EC, %**	Yes	19.4	17.1	23.5	χ^2^ = 34.78, p < 0.001, V = 0.077	16.6	25.5	23.0	χ^2^ = 57.95, p < 0.001, V = 0.100	18.1	23.9	χ^2^ = 21.10, p < 0.001, V = 0.060
**Current NRT or EC, %**	Yes	25.4	22.6	30.5	χ^2^ = 44.64, p < 0.001, V = 0.087	22.3	31.6	30.6	χ^2^ = 56.02, p < 0.001, V = 0.098	23.5	32.4	χ^2^ = 41.13, p < 0.001, V = 0.084
**Weekly spend Mean (SD)** ^b^	**£**	22.6 (17.1)	23.3 (17.3)	21.4 (16.7)	F = 15.30, p < 0.001	23.3 (17.3)	21.5 (16.8)	20.8 (16.2)	F = 8.75, p < 0.001	22.7 (17.1)	22.3 (17.0)	F = 0.57, *p* = 0.45
**Weekly spend Median** ^b^	**£**	20	20	16	p < 0.001	20	17	15	p < 0.001	20	17	*p* = 0.33

*MTSS* Motivation to stop smoking

^a^The provided third option was not used by anyone in the included sample

^b^Type of cigarette missing unweighted *n* = 164, weekly spend missing unweighted *n* = 216

^c^The median number of quit attempts was 0 for all groups; the mean number of quit attempts overall was 0.59 (SD = 3.10), this was similar for those with (M = 0.67; SD = 3.00) and without diagnosis (M = 0.55, SD = 3.15; F(1,6316) = 2.13, *p* = 0.14), with (M = 0.71, SD = 2.82) and without past-year treatment (M = 0.56, SD = 3.17; F(1,6316) = 2.42, *p* = 0.112) and differed slightly across levels of distress (minimal: M = 0.51, SD = 3.08, moderate: M = 0.76, SD = 3.55, serious: M = 0.70, SD = 1.66; F(2,6315) = 3.93, p = 0.02)

### Smoking

About 7% of the sample reported having stopped smoking completely in the past year. Most participants were daily cigarette smokers, and there was little difference between those with and without mental health problems. Overall, about half smoked roll-your own exclusively (44.1%) or alongside manufactured cigarettes (5.4%) and this was more common among cigarette smokers with mental health problems. The vast majority of past-year smokers experienced at least some urges to smoke although few had high heaviness of smoking ratings. Stronger urges and heavier smoking were more common among those with mental health problems (Table 1).

Current cigarette smokers with data on spend available (*n* = 5421) on average spent £22.61 (SD = 17.09) per week on tobacco or cigarettes (median £20). This was lower among those with any diagnosis or distress, but not by treatment (Table 1), likely in part due to higher rates smoking roll-your own.

### Harm reduction

Overall, 46.1% of smokers were cutting down, 19.4% were using e-cigarettes and 8.5% nicotine replacement therapy (Table 1). All three harm reduction behaviours were more common among smokers with a mental health problem, albeit all with Cramer’s V ≤ 0.1 (Table 1). About one in seven (14.4%) reported motivation to stop smoking within the next three months. This was slightly more common among those with any indicator of mental health problems, with Cramer’s V below 0.1. (Table 1). In the last year, 12.9% had achieved at least one month of abstinence. This did not differ by ever diagnosis or past-year treatment but by level of distress; those with minimal distress were least likely and those with moderate distress most likely to have achieved a month of abstinence, again with Cramer’s V below 0.1 (Table 1).

### Quit attempts

Just under a third (32.5%) of those who smoked in the past year had made at least one quit attempt in that time. In unadjusted analysis, prevalence of having made at least one quit attempt was higher among past-year smokers with any diagnosis (36.2, 95% CI: 34.2–38.1; unadjusted OR = 1.36, 95% CI: 1.22–1.53, *p* < 0.001) than among those without diagnosis (30.4, 95% CI: 29.0–31.8). Prevalence of having made at least one quit attempt increased with distress, from 29.6% (95% CI: 28.2–31.0) among those with no or minimal distress through 37.7% (95% CI: 35.3–40.1; unadjusted OR = 1.45, 95% CI: 1.28–1.64, *p* < 0.001) among those with moderate distress to 38.8% (95% CI: 35.0–42.7; unadjusted OR = 1.54, 95% CI: 1.28–1.84, p < 0.001) among those with serious distress. Treatment in the past year was also associated with higher prevalence of having made a quit attempt (38.3%; 95% CI: 35.7–40.8; unadjusted OR = 1.44, 95% CI: 1.27–1.64, p < 0.001) compared with not having had treatment (30.8%; 95% CI: 29.6–32.1). The strength of these associations remained very similar in adjusted analysis (Table 2). When including only current smokers, smokers with any diagnosis, past-month distress or past-year treatment were also more likely to have made a quit attempt than smokers without these indicators of a mental health problem (Table 2).

**Table 2:** Adjusted associations with having made at least 1 quit attempt in the past year by mental health diagnosis, level of past-month distress and past-year treatment for a mental health problem (unweighted data)

		***n*** = 6071 past-year cigarette smokers				***n*** = 5506 current smokers			
		AOR	95% CI	95% CI	p	AOR	95% CI	95% CI	p
**Any mental health diagnosis**	No	1				1			
	Yes	**1.32**	**1.17**	**1.48**	**< 0.001**	**1.25**	**1.09**	**1.43**	**0.001**
**Past-month distress**	None/minimal	1				1			
	Moderate	**1.41**	**1.24**	**1.60**	**< 0.001**	**1.35**	**1.17**	**1.57**	**< 0.001**
	Serious	**1.51**	**1.25**	**1.81**	**< 0.001**	**1.51**	**1.22**	**1.87**	**< 0.001**
**Past-year treatment**	No	1				1			
	Yes	**1.41**	**1.24**	**1.61**	**< 0.001**	**1.37**	**1.18**	**1.60**	**< 0.001**

Separate models for 1. Any mental health diagnosis, 2. Level of past-month distress, 3. Past-year treatment for any mental health problem

For past-year cigarette smokers adjusted for age, gender and occupational grade

For current smokers adjusted for age, gender, occupational grade, motivation to stop smoking and type of cigarette

## Discussion

Different indicators of mental health problems showed that substantial proportions of past-year smokers in England have mental health problems. Just over a third reported ever having had a diagnosis of a mental health problem and around 60% of them had received treatment in the last year, suggesting an ongoing issue which was also indicated by just over a third experiencing moderate or serious mental distress in the past month. Those with an indication of a mental health problem were more highly dependent and more likely to smoke roll-your-own cigarettes. Cigarette smokers with a mental health problem were however more likely to be motivated to stop smoking and to engage in harm reduction behaviours such as use of e-cigarettes or NRT. Past-year smokers with mental health problems were more likely to have made at least one quit attempt in the past year and this remained true when including only current smokers (i.e. where any quit attempts had been unsuccessful). Yet, there was little difference by mental health in having achieved at least short-term abstinence among all past-year smokers, suggesting that quit attempts may be less successful in those with mental health problems.

The prevalence of mental health problems found in this population survey is in line with previous estimates that 30% of smokers in the UK have evidence of mental disorder [5] and an estimate that 27.4% of adults in England have ever been diagnosed with a common mental health problem (generalised anxiety disorder, depressive episode, obsessive compulsive disorder, phobias, panic disorder [36], omitting groups of less common diagnoses included in the present estimate). The level of moderate and severe distress found in past-year smokers in England is similar to those from a household survey in the US which found that in 2015, 25.8% of current cigarette smokers had moderate and 9.4% serious distress in the past month [12]. Both a US survey [12, 30] and the present survey found that those with psychological distress were more likely to have made at least one quit attempt in the past year. The present finding that those with a mental health problem are at least as likely to be motivated to stop smoking is in line with earlier findings which used different measures to measure related constructs [4, 30, 37]. Previous research from the US found that current cigarette smokers with mental health problems were not statistically more likely to be using electronic cigarettes, this is in contrast to the present findings [38, 39].

The present study had several limitations. Timing or causality of events cannot be established; it could for example be argued that an unsuccessful quit attempt in the past-year could have increased distress and even increased the need for treatment for a mental health problem. However, associations were very similar for the different indicators of mental health problems, making this direction of causality appear less likely. All measures were self-reported; more comprehensive instruments may have differed in their detection of mental health problems. Because the survey is a household survey, it excludes people who are too unwell to take part or in healthcare institutions, thereby excluding those with the most severe mental health problem. Because respondents were asked to select the single option that best describes their smoking, use of other tobacco products such as pipes, cigars or shisha remained unreported among cigarette smokers. However, a strength is that data came from a long-standing established population survey and a sample representative of the adult population in England.

The present study is the first to provide detailed population-level evidence on motivation to quit smoking, attempts to do so and harm reduction behaviours of smokers in England by mental health status. It is also the first to report that smokers with a mental health problem are more likely to engage in harm reduction behaviours compared to those without.

Some implications for policy and clinical practice can be drawn from the findings. The relatively higher levels of motivation, attempts to stop smoking and harm reduction behaviours such as e-cigarette use among smokers with mental health problems compared to those without should be capitalised on by clinicians to reduce the burden from smoking. Specific challenges such as higher dependence on tobacco need to be addressed by making effective support available. A combination of behavioural support and medication is most effective in the wider population of smokers and has been shown to be effective for smokers with severe mental illness [14, 15] and for smokers with and without mental health problems making quit attempts [40]. In quit attempts and accompanied by behavioural support, e-cigarettes have been shown to be more effective than NRT [41]. In the Smoking Toolkit Study, they were also associated with increased success rates for smokers with and without mental health problems [40]. Additionally, smaller studies in smokers with severe mental illness who were not interested in quitting found that substantial proportions reduced their smoking by at least 50% when supplied with e-cigarettes [42, 43]. Clinicians should ensure that patients (both inpatient and community) have their smoking status identified and recorded in health care records. All smokers irrespective of their mental health status should be advised about the most effective way to quit and either provided with treatment or referred for specialist stop smoking support [44]. Ensuring harm reduction strategies are accessible should also be considered. Recently in England, the National Health Service has committed to funding tobacco treatment services for all smokers admitted to hospital, including the option to switch to e-cigarettes for mental health patients [45].

Future research needs to assess associations for specific mental health problems or groups of problems should also be assessed in addition to a catch-all category of any diagnosis.

## Conclusions

About a third of smokers in England have mental health problems. This population is more likely to be young, female and from lower socio-economic groups. They are more dependent on tobacco smoking but also more likely to engage in harm reduction behaviours, more motivated and more likely to attempt to stop smoking. Interventions should address their increased dependence but also leverage the higher prevalence of harm reduction behaviours, motivation to stop smoking and attempts to stop smoking in this population.

## Supplementary information

**Additional file 1.** Supplementary file 1: Table S1. Comparison of past-year smokers who completed the mental health information and those who terminated the survey prior to the section or responded don’t know or prefer not to say.

**Additional file 2.** Supplementary file 2: Table S2. Weighted prevalence of individual diagnosis and past-year treatment for each diagnosis.

## Data Availability

All authors have had access to the data. The datasets analysed during the current study are available from the corresponding author on reasonable request.

## Abbreviations

ANOVA: Analysis of variance
CI: Confidence interval
NRT: Nicotine replacement therapy
OR: Odds ratio
UK: United Kingdom
US: United States of America
