# Supplementary material for: Mental health, smoking, harm reduction and quit attempts – a population survey in England
Source: BMC Public Health. 2020 Aug 14;20:1237. doi: 10.1186/s12889-020-09308-x (PMC7427923; doi:10.1186/s12889-020-09308-x)
Supplement: Supplementary file 1 — Additional file 1. Supplementary file 1: Table S1. Comparison of past-year smokers who completed the mental health information and those who terminated the survey prior to the section or responded don’t know or prefer not to say. [file 12889_2020_9308_MOESM1_ESM.docx]

# Mental health, smoking, harm reduction and quit attempts – a population survey in England

## Leonie S. Brose, Jamie Brown, Debbie Robson, Ann McNeill

### Supplementary material

**Table S1. Comparison of past-year smokers who completed the mental health information and those who terminated the survey prior to the section or responded don’t know or prefer not to say.**

Weighted percentages, unweighted n=7,465.

| **Characteristic** | | **Not completed, %** | **Completed, %** | **chi-square, p (asymptotic)** | **Cramer’s V** |
| --- | --- | --- | --- | --- | --- |
| **Age** | 16-24 | 13.2 | 86.8 | **16.26, 0.006** | **0.046** |
|  | 25-34 | 15.1 | 84.9 |  |  |
|  | 35-44 | 15.7 | 84.3 |  |  |
|  | 45-54 | 16.4 | 83.6 |  |  |
|  | 55-64 | 18.9 | 81.1 |  |  |
|  | ≥65 | 17.0 | 83.0 |  |  |
| **Gender ^1^** | Men | 15.8 | 84.2 | 0.04, 0.85 |  |
|  | Women | 15.7 | 84.3 |  |  |
| **Occupational grade** | AB | 15.0 | 85.0 | 2.63, 0.621 | 0.018 |
|  | C1 | 16.2 | 83.8 |  |  |
|  | C2 | 14.9 | 85.1 |  |  |
|  | D | 16.1 | 83.9 |  |  |
|  | E | 16.6 | 83.4 |  |  |
| **Smoking** | Daily cigs | 15.0 | 85.0 | **29.30, <0.001** | **0.061** |
|  | Non-daily cigs | 21.8 | 78.2 |  |  |
|  | Stopped last year | 14.3 | 85.7 | 0.16, 0.922 | 0.005 |
| **Type of cigarette** | Manufactured | 15.5 | 84.5 |  |  |
|  | Roll-your-own | 15.2 | 84.8 |  |  |
|  | Mix | 15.3 | 84.7 |  |  |
| **Urges to smoke** | None | 16.6 | 83.4 | 6.17, 0.29 | 0.028 |
|  | 1 | 17.1 | 82.9 |  |  |
|  | 2 | 14.8 | 85.2 |  |  |
|  | 3 | 15.3 | 84.7 |  |  |
|  | 4 | 17.7 | 82.3 |  |  |
|  | 5 | 16.7 | 83.3 |  |  |
| **HSI** | Low (<4) | 15.5 | 84.5 | 0.72, 0.40 | 0.01 |
|  | High (4+) | 16.6 | 83.4 |  |  |
| **≥1 quit attempt** | Yes | 15.8 | 84.2 | 0.34, 0.56 | 0.007 |
| **Abstinence ≥1 month** | Yes | 17.8 | 82.2 | 3.51 0.061 | 0.021 |
| **Current cigarette smokers** | | |  |  |  |
| **MTSS** | In ≤3 months | 17.3 | 82.7 | 1.77, 0.184 | 0.016 |
| **Cutting down** | Yes | 16.5 | 83.5 | 1.81, 0.17 | 0.016 |
| **Current NRT** | Yes | 20.3 | 79.7 | **12.24, <0.001** | **0.040** |
| **Current EC** | Yes | 16.4 | 83.6 | 0.63, 0.43 | 0.009 |

^1^ 2 respondents who chose a third option did not complete the mental health information
