# Supplementary material for: Mental health, smoking, harm reduction and quit attempts – a population survey in England
Source: BMC Public Health. 2020 Aug 14;20:1237. doi: 10.1186/s12889-020-09308-x (PMC7427923; doi:10.1186/s12889-020-09308-x)
Supplement: Supplementary file 2 — Additional file 2. Supplementary file 2: Table S2. Weighted prevalence of individual diagnosis and past-year treatment for each diagnosis. [file 12889_2020_9308_MOESM2_ESM.docx]

# Mental health, smoking, harm reduction and quit attempts – a population survey in England

## Leonie S. Brose, Jamie Brown, Debbie Robson, Ann McNeill

### Supplementary material

**Table S2.** **Weighted prevalence of individual diagnosis and past-year treatment for each diagnosis.** Weighted percentages, unweighted n=6,071.

| **Diagnosis** | **Prevalence, %** | **Past-year treatment, %** |
| --- | --- | --- |
| Depression | 27.2 | 62.2 |
| Anxiety | 20.6 | 59.9 |
| Panic Disorder or Phobia | 5.5 | 45.7 |
| Alcohol Misuse or Dependence | 3.9 | 20.7 |
| Post-Traumatic Stress Disorder (PTSD) | 3.4 | 43.6 |
| Drug Use or Dependence | 3.0 | 27.1 |
| Eating Disorder | 2.6 | 18.7 |
| Obsessive Compulsive Disorder | 2.4 | 31.9 |
| Personality Disorder | 2.2 | 62.4 |
| Psychosis | 1.6 | 64.1 |
| Attention Deficit Hyperactivity Disorder (ADHD) | 1.3 | 25.2 |
| Problem Gambling | 0.4 | 7.9 |
